# Supplementary material for: Mutations in PCYT2 disrupt etherlipid biosynthesis and cause a complex hereditary spastic paraplegia
Source: Brain. 2019 Oct 22;142(11):3382–97. doi: 10.1093/brain/awz291 (PMC6821184; doi:10.1093/brain/awz291)
Supplement: awz291_Supplementary_Data [file awz291_supplementary_data.zip › awz291-Suppl_data/Supplementary_Data4.zip!/Supplementary information 3 - fibroblast lipidomics.zip!/Fibroblast lipidomics/Controle vs Patient1/Statistical plots and data/PCA.pdf]

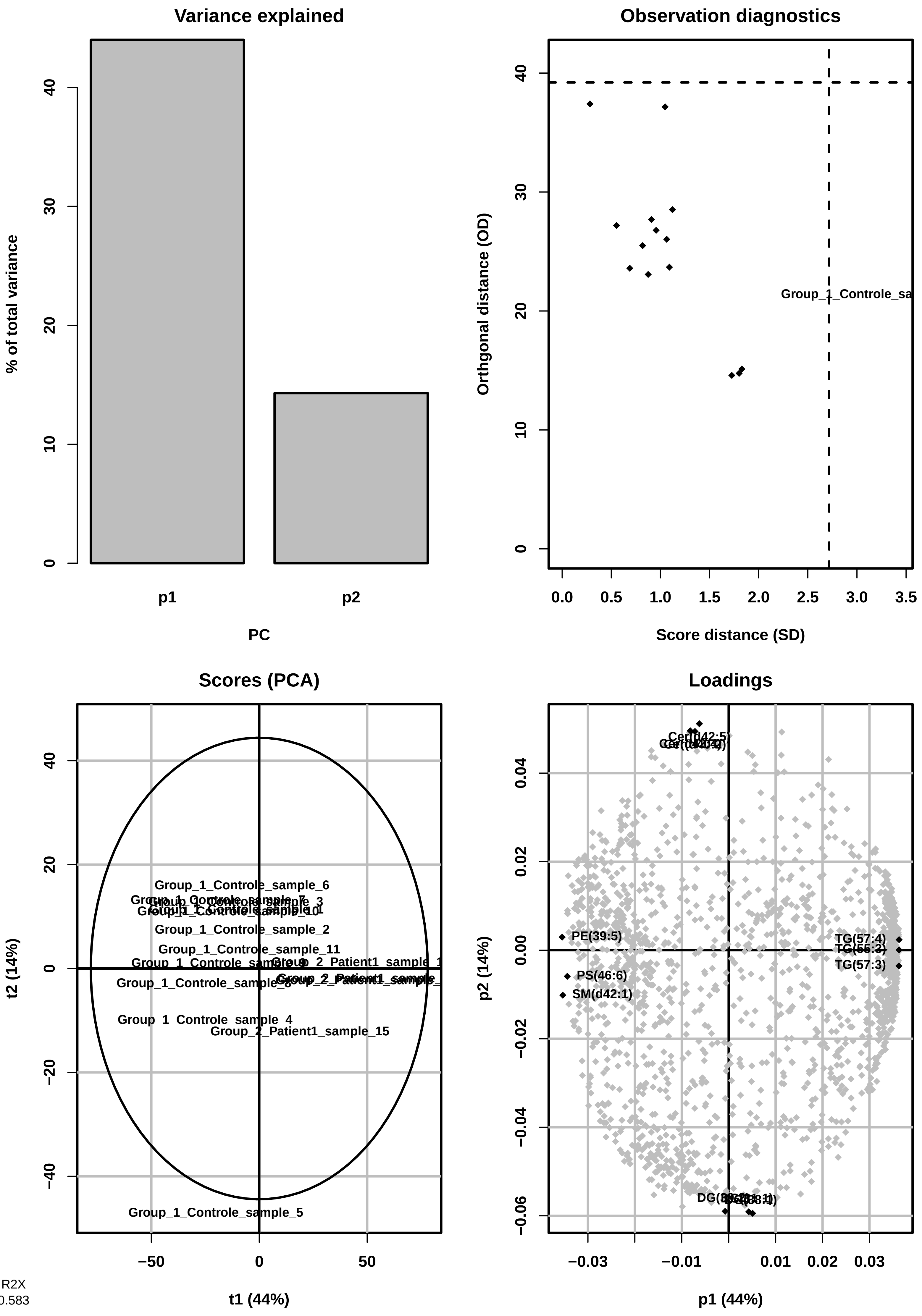

Variance explained
% of total variance
PC
p1
p2
Observation diagnostics
Orthgonal distance (OD)
Score distance (SD)
Group_1_Controle_sa
Scores (PCA)
t1 (44%)
t2 (14%)
Group_1_Controle_sample_6
Group_1_Controle_sample_2
Group_1_Controle_sample_11
Group_1_Controle_sample_8
Group_1_Controle_sample_4
Group_2_Patient1_sample_15
Group_1_Controle_sample_5
Loadings
p1 (44%)
p2 (14%)
PE(39:5)
PS(46:6)
SM(d42:1)
TG(57:4)
TG(57:3)
R2X
0.583
